# Attachment insecurities, caregiver burden, and psychological distress among partners of patients with heart disease

**DOI:** 10.1371/journal.pone.0269366

**Published:** 2022-09-19

**Authors:** Simone Zofia Laflamme, Karen Bouchard, Karolina Sztajerowska, Kathleen Lalande, Paul S. Greenman, Heather Tulloch

**Affiliations:** 1 Division of Cardiac Prevention and Rehabilitation, Prevention and Rehabilitation Centre, University of Ottawa Heart Institute, Ottawa, Ontario, Canada; 2 Faculty of Social Sciences, School of Psychology, University of Ottawa, Ottawa, Ontario, Canada; 3 Institut du Savoir Montfort, Ottawa, Ontario, Canada; 4 Department of Psychoeducation and Psychology, Université du Québec en Outaouais, Gatineau, Québec, Canada; PLOS (Public Library of Science), UNITED KINGDOM

## Abstract

Caregiver psychological distress (i.e., depression and anxiety) is harmful to both caregiver and patient. Different affect-regulation strategies associated with attachment orientations may impact a caregiver’s perception of their caregiving role as a burden, thereby contributing to their psychological distress. The aim of the present investigation was to examine the links among attachment orientations, caregiver burden, and psychological distress in a cardiac context. Participants (*N* = 181, *M*_*age*_ = 61.79, *SD* = 10.49; *males* = 24.7%) were romantic partners of patients with heart disease (i.e., informal caregivers) who completed validated questionnaires. The majority of caregivers had partners with coronary artery disease (*n* = 127, 70. 2%). 66.3% of caregivers reported low burden, 87.6% reported low levels of depression and 89.9% reported low levels of anxiety. The mean anxious attachment score was 2.74 (*SD* = 1.37) and the mean avoidant attachment score was 2.95 (*SD* = 1.26). Four mediation analyses were run using PROCESS macro for IBM SPSS (version 26). Statistical models showed that the relationships between attachment anxiety and psychological distress were mediated by caregiver burden [*ab*_*anxiety*_= 0.15, 95% *C*.*I*. (0.04, 0.29); *ab*_*depression*_ = 0.15, 95% *C*.*I*. (0.05, 0.28)] and that attachment avoidance was not a significant covariate (*cv*_*anxiety*_ = −0.02, *p*>0.05; *cv*_*depression*_ = 0.40, *p*>0.05). The relationships between attachment avoidance and psychological distress were also mediated by caregiver burden [a*b*_*anxiety*_ = 0.23, 95% *C*.*I*. (0.10, 0.42); *ab*_*depression*_ = 0.21, 95% *C*.*I*. (0.09, 0.37]with attachment anxiety as a significant covariate (*cv*_*anxiety*_ = 1.09, *p*<0.001; *cv*_*depression*_ = 1.09, *p*<0.001). Interventions for caregivers reporting attachment insecurity and burden should be explored to potentially lessen caregiver distress as they support their partners with heart disease.

## Introduction

Cardiovascular disease is an umbrella term that encompasses heart and vascular diseases. It was the leading cause of death for non-communicable diseases worldwide in 2017, accounting for 17.8 million deaths. Heart disease, in particular, largely contributed to this high rate of mortality [1]. The chronic nature of heart disease requires patients to make adjustments to many areas of their life; to do so, many receive support from an “informal caregiver,” (i.e., a spouse, life partner, child, relative, or friend who provides unpaid care to people with a serious illness or other chronic conditions) [2,3]. Caregivers contribute to patient recovery in many ways, including advocating for their loved ones, providing emotional encouragement, and helping with self-care routines [4–6]. In the cardiac context, the latter can include a wide range of activities such as monitoring the patient’s blood pressure, encouraging or participating in exercise, as well as coordinating and attending medical appointments [4]. Caregiver involvement is vital to patient recovery. For example, assisting their loved one adhere to medical recommendations can lead to improved health. Lacking this support and related adherence can lead to health deterioration and hospital readmission [4,5,7,8]. Empirical evidence demonstrates that caregiving may result in fewer symptoms of anxiety and depression and better adjustment to life with heart disease in comparison to patients with limited family support [9,10]. Caregivers themselves may also derive a sense of satisfaction, reward, and purpose from caregiving [11]. Further, caregivers’ endeavors reduce the burden on the health care system [5]; annual savings to the American health care system are estimated to be $350 billion [12]. This positive influence on the patient’s health, however, may come at a personal cost to the caregiver and it can take a toll on intimate relationships when the caregiver is a spouse or life partner [2,5,11,13]. For instance, caregivers commonly experience psychological distress and “caregiver burden,” which is defined as the physical, social, economic, and emotional hardship suffered by those in a caregiving role [14].

### Caregiver burden in a cardiac context

Caregivers of patients with heart disease represent a subgroup of caregivers who often feel physically, emotionally, and psychologically burdened by their role [5,11,13,15]. They may endure sleep disruption, high rates of fatigue, as well as an elevated risk of heart disease and mortality themselves [5,11,13,16]. Their preoccupation with caring for the patient can lead to the neglect of their own health and poor health behaviors, such as poor eating habits and lack of exercise [13]. Furthermore, caregivers’ ability to maintain social and family relationships may be strained while they perform their caregiving role, which can lead to feelings of isolation and disconnection [11]. When the caregiver is the romantic partner of the patient, the changes that occur within the couple relationship as a result of the disease (e.g., sexual concerns, communication difficulties, adjustment to a new lifestyle) can also be considered a burden [2,6]. Financially, there may be a reduction in household income during the time of increased caregiving leading to feelings of burden and distress [11]. Caregivers of patients with heart disease are also known to experience psychological distress as a result of their partner’s heart disease [2,17–19].

### Psychological distress among caregivers of patients with heart disease

Psychological distress (i.e., depression and anxiety) appears to be particularly salient in caregivers of patients with heart disease surpassing symptom levels of the general population and even the patients themselves [9,20–24]. Approximately 25–30% of caregivers experience symptoms of anxiety six months post cardiac event and 25% continue to experience these symptoms one year later [25,26]. Interestingly, Singh et al. [27] demonstrated that anxiety two months post-surgery may be ameliorated through anticipation of caregiving demands before surgery. In regard to depression, 19–43% of caregivers of patients with heart disease experience symptoms during hospitalization, and 14–38% experience these symptoms within one-year post-hospitalization [24,25]. Psychological distress among these caregivers is related to poor quality of life, difficulties with daily caregiving, low perceived control, negative relationship quality with the patient, decreased functional ability, and inhibited ability to retain vital information regarding patient discharge and care [28–30]. Further, caregivers’ psychological distress is linked to increased hospitalization of the patient [5]. Increased levels of psychological distress in the caregiver also produce undesirable psychological and behavioral consequences for the patient such as symptoms of anxiety and depression, and poor self-care which, in turn, contribute to increased hospitalization [4,6,28,30–32].

### Attachment theory overview

Attachment theory may shed some light on the experience of caregiver burden and caregiver psychological distress [5,11,13]. Attachment theory posits that individuals develop, starting in infancy, a habitual way of relating to others through interactions with their primary caregivers [33]. According to attachment theory, all human beings have an innate biological drive to engage in proximity-seeking behaviors (known as “attachment behaviors”) with emotionally available attachment figures when distressed. The function of proximity-seeking is to regulate distress and emotions in the presence of a threat. During childhood, parents tend to be primary attachment figures, that is, those to whom children usually turn for proximity and emotional support. In adulthood, romantic partners generally occupy this role and patterns of attachment behaviors, emotions, and relational expectations learned in childhood play out in the couple relationship [34,35].

Attachment theory posits that individuals develop an attachment orientation depending on their position along dimensions of attachment-related avoidance and anxiety [36]. Adults low on attachment anxiety and avoidance are considered securely attached, meaning that they have a positive representation of themselves (i.e., “I am lovable and competent”) and others (i.e., “people will be there for me when I need them”). Individuals with a stable sense of attachment security have effective affect regulation strategies known as primary attachment strategies [37]. They tend to recognize their distress, are confident in their ability to cope with the threat and seek out emotional support from trusted others. They appropriately assess the severity of a threat, communicate their emotions, and view life more positively [37].

Adults with avoidant and anxious attachment (i.e., attachment insecurities) employ secondary attachment strategies that are less effective ways of regulating one’s emotions than are the methods employed by those with a stable sense of attachment security [37,38]. Those with attachment-related avoidance use deactivating strategies that may be a result of experiences with a childhood attachment figure that was neglectful or punitive in response to proximity-seeking behaviors [37,39,40]. As a result, individuals with attachment-related avoidance distrust others, including their attachment figures [39,40]. Deactivating strategies include denying attachment needs by supressing emotions related to threats or vulnerability and refraining from proximity-seeking behaviours as well as interdependence [39,41].

Those with attachment-related anxiety engage in hyperactivating strategies that may be a result of experiences with a childhood attachment figures who provided inconsistent responses to proximity-seeking behaviours [39,40]. As a result, individuals who score high on this attachment orientation worry that their attachment figure will not respond to their proximity-seeking behaviors. Further, they fear abandonment since they feel vulnerable without a consistent attachment figure to co-regulate their emotions and are unsure of their ability to regulate distress on their own [42]. Hypervigilant strategies are intense responses to perceived threats that may be disproportional to the actual danger [39]. Hyperactivating strategies such as exagerating a threat and one’s own vulnerability, hyper-focusing on internal distress (e.g., rumination and overattention to physiological symptoms of distress), and escalating negative emotions through self-destructive behaviors are employed in order to increase attachment figure responsivess [37]. These individuals create a perpetual cycle of distress and become angry or resentful when their attachment figure does not respond to proximity-seeking behaviours [37,38].

### Attachment insecurities, caregiver burden, and psychological distress

Caregivers of patients with heart disease face the potential loss of their attachment figure and practical challenges that arise due to their caregiving role [5,11,13,15]. An individual with anxious attachment will be likely to intensify their affective response to these threats using hyperactivating strategies in order to elicit a response from their attachment figure [37,43,44]. Hyperactivating strategies may contribute to caregiver burden because they position caregivers with anxious attachment to perceive the challenges of caregiving as more severe hardship than caregivers with secure attachment, who would be more likely to appropriately assess the severity of a threat and their ability to cope with it [37,45–47]. The intensified experience of caregiver burden may then increase the caregiver’s experience of psychological distress [25,48].

In contrast, caregivers with attachment avoidance would tend to deactivate their affective response to the potential loss of a partner and the practical challenges of their caregiving role [38,41]. This strategy may lessen their experience of caregiver burden because it could allow them to address the practical challenges of caregiving while inhibiting negative affective responses that may worsen their perception of the severity of the burden [43,44,47]. Caregivers with attachment avoidance may, therefore be less likely to report their negative affect than caregivers with attachment anxiety or attachment security who acknowledge their negative affect [49].

In the cardiac population, a thorough literature review revealed only one study that focused on the relationship between caregiver burden, attachment insecurities, and psychological distress among patients with heart disease and their caregivers. Vilchinsky et al. [48] investigated how the caregiver’s attachment orientation moderated the relationship between caregiver burden and symptoms of depression in women caring for their romantic partner with Acute Coronary Syndrome. The authors found that attachment anxiety strengthened the relationship between caregiver burden and symptoms of depression six months after the cardiac event. In other words, a stronger association between caregiver burden and depression was found when caregivers had higher levels of attachment anxiety. Attachment anxiety in cardiac caregivers thus appears to worsen depression. In contrast, attachment avoidance was not a significant moderator [48].

The present study expands our current understanding of attachment insecurities, caregiver burden and psychological distress by using mediation analyses to investigate new relationships among these variables. As noted, attachment orientation in caregivers of patients with heart disease may influence caregiver burden and, thereby caregiver psychological distress. For these reasons, the principal aim of this study was to investigate if attachment orientation was indirectly related to psychological distress through its relationship with caregiver burden. We hypothesize that attachment anxiety will increase caregivers’ experience of burden, subsequently leading to increased psychological distress. In contrast, attachment avoidance will limit caregivers’ experience of burden and, therefore, lower their psychological distress.

## Methods

### Participants and procedure

The current study is part of a larger cross-sectional, observational study of cardiac patients and their partners (i.e., caregivers). Outpatients from a large cardiac centre who were in a romantic relationship were contacted in person or by phone and screened for eligibility. If eligible, research staff met with the patient (and the caregiver, if present) to further explain the study, obtain written and informed consent and administer the questionnaires. If only the patient was present at the consent appointment, the consent form and questionnaires for the caregiver were given to the patient to be filled out at home and contact information was provided should the caregiver have any questions. Participants were instructed to complete their questionnaires independently. Questionnaires were returned on-site or by mail using prepaid return envelopes. Data were collected between January and December, 2019. Participants were not compensated for their participation. Data from the caregivers is presented in this paper. This study was approved by the Ottawa Health Science Network Research Ethics Board and all participants provided written and informed consent.

**Fig 1:**
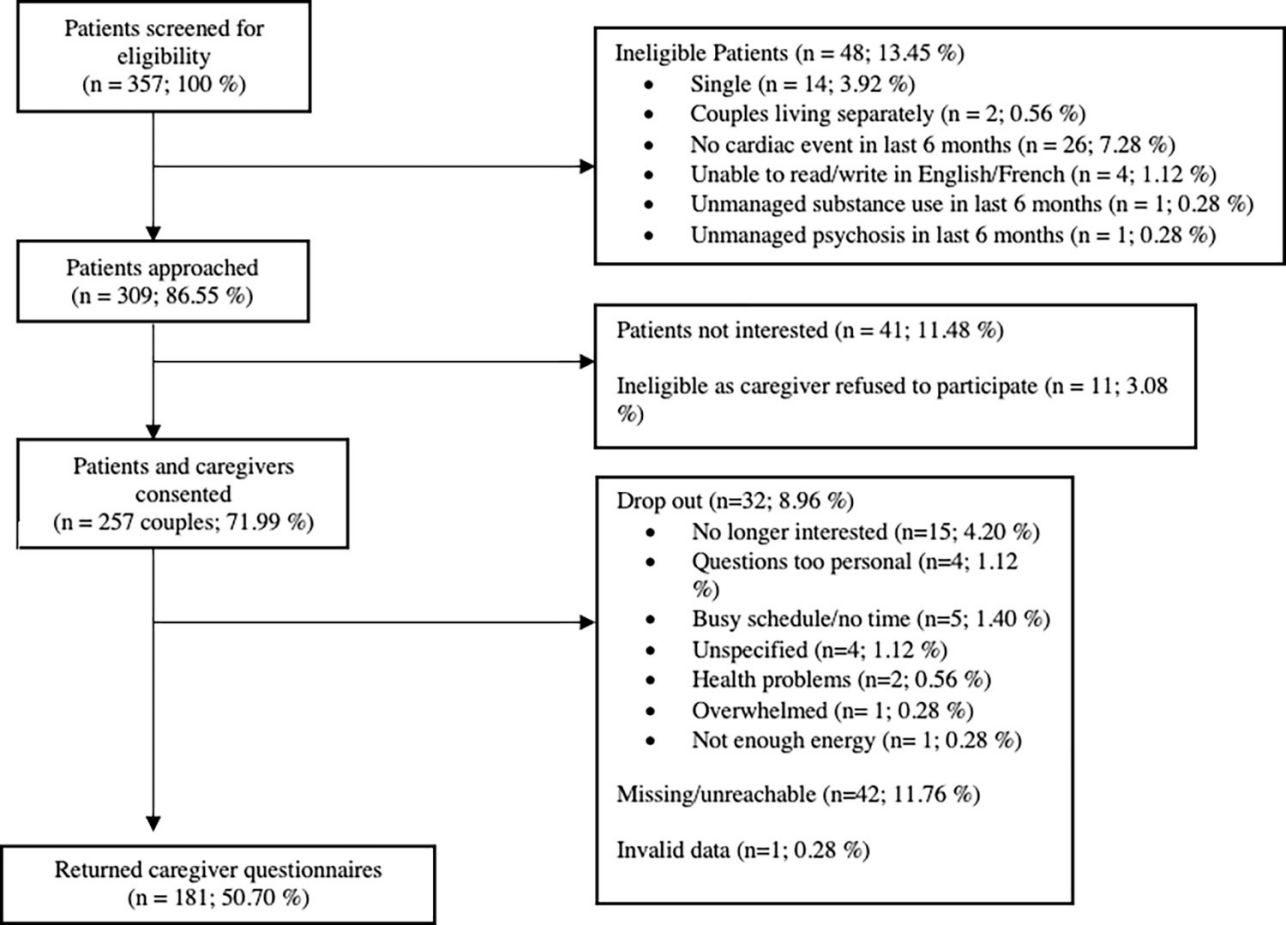
Participant flow.

Three hundred and nine patients who consented to research participation at the cardiac center were screened and deemed eligible to participate. Of these, 257 patients and their partners gave their consent to participate. Thirty-two couples dropped out, 42 couples did not return their questionnaires or were unreachable, one couple had invalid data (i.e. they completed their questionnaires together), and one couple had a partner who passed away. As a result, the final sample included 181 caregivers. Please see Fig 1.

### Inclusion and exclusion criteria

Inclusion criteria were as follows: (a) participants experienced directly or indirectly (i.e. spouse) a cardiac event in the last six months, (b) participants were in a couple relationship (married, common-law, or in a committed relationship for ≥ one year), (c) participants had been living in the same household for at least six months, (d) participants were 18 years of age or older, (e) participants were able to read and/or speak English or French, (f) participants and their partner were willing to provide informed consent. Exclusion criteria were as follows: (a) active substance abuse, and (b) unmanaged mental health symptoms (e.g., active psychosis, suicidal ideation) in the last six months.

### Measures

#### Demographic and clinical information

Demographic characteristics reported by participants were age, gender, ethnic origin, employment status, educational level, total income, marital status, and years in their relationship. Medical information (both mental and physical health) was self-reported; cardiac diagnosis of the patient was verified by their medical chart.

#### The Experiences in Close Relationships Scale-12

The *Experiences in Close Relationships Scale-12* (ECR-12) was used to measure attachment orientations among adults in couple relationships [50]. This 12-item scale assesses attachment using the dimensions of attachment related anxiety and avoidance. A sample item from the attachment anxiety subscale is “I worry that romantic partners won’t care about me as much as I care about them” and a sample item from the attachment avoidance subscale is “I don’t feel comfortable opening up to romantic partners.” Responses are indicated on a 7-point Likert scale ranging from 1 (strongly disagree) to 7 (strongly agree). Subscales were calculated by reverse coding the appropriate items and averaging the respective items for each subscale. Low scores on both subscales (anxious and avoidant subscales) indicate higher levels of secure attachment [50]. This measure is valid [50] and had an acceptable Cronbach alpha of 0.74 in the present study.

#### The Generalized Anxiety Disorder Scale -7

The *Generalized Anxiety Disorder Scale-7* (GAD-7) was used to assess symptoms of anxiety (e.g., trouble relaxing, worrying too much about different things). Responses are indicated on a 4-point Likert scale with options ranging from 0 (not at all) to 3 (nearly every day). Cut-off scores are five, 10 and 15 for mild, moderate and severe levels of anxiety, respectively [51]. This measure is valid [51] and it has been used in the general population as well as a cardiac population [51–53].

#### Patient Health Questionnaire– 9

The *Patient Health Questionnaire* (PHQ-9) was used to assess symptoms of depression (e.g., little interest or pleasure in doing things; feeling down, depressed, or hopeless). Responses are indicated on a 4-point Likert scale with options ranging from 0 (not at all) to 3 (nearly every day). This nine-item scale has cut-off scores of five, 10, 15 and 20 for mild, moderate, moderately severe and severe depression [54]. This measure is valid and has been used in cardiac populations [52–54].

#### Zarit Burden Interview– 22

The *Zarit Burden Interview* (ZBI-22) was used to assess caregiver burden. It is a 22-item questionnaire that assesses both subjective and objective experience of burden [55]. Sample items are “Do you feel that your relative asks for more than he/she needs?” and “Do you feel angry when you are around your relative?” Responses are indicated on a 5-point Likert scale ranging from 0 (never) to 4 (nearly always). Caregivers that score 17 or higher are considered to experience high levels of burden [55]. This scale was originally created to assess burden in caregivers of patients with dementia but has since been validated in patients with heart failure [55]. In the present study, Cronbach’s alpha was excellent (0.91).

### Statistical analysis

A priori power analyses were conducted using G*power. Little’s [56] “Missing Completely At Random” (MCAR) test was performed to identify if data were missing completely at random. The Expectation-Maximization technique was used to replace missing data and a sensitivity analysis was run to ensure that all significant relationships from the imputed data set were also significant from the non-imputed data set. Two sets of parametric tests were run: one for both models with anxiety as the outcome variable and one for both models with depression as the outcome variable. To start, participants were removed if they were identified as outliers on two or more of the following tests: Mahalanobis distance, Cook’s distance and Leverage. Further, Pearson correlations were computed to test multicollinearity. Multivariate normality, linearity, homogeneity and homoscedasticity assumptions were also analyzed as part of the parametric tests. The caregiver’s history of depression, anxiety and physical health problems were explored as potential covariates to caregiver burden through point-biserial correlations; all three potential covariates were categorical in nature (i.e. yes, no, prefer not to answer). Finally, descriptive statistics were used to analyze sample characteristics.

As per Kane and Ashbaugh [57], four mediation analyses were conducted in order to test whether caregiver burden mediated the relationship between attachment orientation and psychological distress. These tests were conducted using PROCESS macro for IBM SPSS (version 26), which centers variables and facilitates mediation analyses using bootstrapping techniques [58]. Two analyses were run with anxiety as the outcome variable (Y). The mediator variable (M) in these analyses was caregiver burden. In the first model, the anxious attachment subscale of the ECR-12 was the independent variable (X) and the avoidant attachment subscale of the ECR-12 was the covariate; vice versa for the second model with anxiety as the outcome variable. The same two analyses were then repeated with depression as the outcome variable (Y). The threshold for significance (alpha) was set at 0.05%.

## Results

### Preliminary analyses

A priori power analyses revealed that, with an estimated r^2^ value of 0.06, 175 participants were needed to achieve a small effect size (*F*^2^ = 0.06) while maintaining 80% power; our sample exceeds this recommended sample size. Little’s MCAR test revealed that the data were missing completely at random (*X*^2^ = 1686.86, *df* = 1698, *p* = 0.50). Missing data ranged from 2.2 to 6.6%. The sensitivity analysis between the imputed and non-imputed data sets did not show a difference in significance in the findings. Due to variables with outliers, five and three outliers were removed in the mediation analyses with anxiety and depression as the outcome variable, respectively. Multicollinearity was acceptable in all analyses (for analyses with depression and anxiety as the outcome variables, respectively, *r*_*ANX*_ = 0.45, *p*<0.01; *r*_*AVD*_ = 0.25, *p*<0.01; *r*_*ANX*_ = 0.40, *p*<0.01; *r*_*AVD*_ = 0.12, *p*>0.05). Multivariate normality, linearity, homogeneity and homoscedasticity were achieved in all analyses. Further, point biserial correlations showed that the caregiver’s history of depression, anxiety and physical health problems were not significantly related to caregiver burden (*r*_*depression*_ = 0.01, *p*>0.05; *r*_*anxiety*_ = 0.06, *p*>0.05; *r*_*physical*_ = −0.003, *p*>0.05).

### Descriptive statistics

The majority of caregivers were female (*n* = 134, 75.3%) and white (91.1%). Their average age was 61.79 years (standard deviation [*SD*] = 10.49), and they were in their current relationship with the patient with heart disease for an average of 33.83 years (*SD* = 14.94). Most caregivers (70.2%) had a partner diagnosed with coronary artery disease (CAD), followed by arrhythmia (17.1%). Approximately 30% of caregivers had high caregiver burden, 8.8% had elevated anxiety scores (i.e., moderate or severe) and 30.4% had moderate to severe depression. Table 1 provides sample characteristics and means and SDs for caregiver burden, caregiver anxiety and depression, and attachment variables.

**Table 1:** Sample characteristics and study variables.

Characteristic	n (%) or M (SD)
	n (%)
**Relationship Status**	
Married	153 (85.0)
Common Law	27 (15.0)
**Ethnicity**	
White	163 (91.1)
Black	3 (1.7)
Latin/Hispanic	2 (1.1)
Asian	7 (3.9)
Middle Eastern	1 (0.6)
Aboriginal	1 (0.6)
Other	2 (1.1)
**Education**	
Elementary school	1 (0.6)
High School	49 (27.4)
College Degree	47 (26.3)
University Degree	81 (45.3)
No Formal Education	1 (0.6)
**Employment**	
Employed Full-Time	55 (30.3)
Employed Part-Time	24 (13.3)
Unemployed	93 (51.4)
Disability Leave	6 (3.3)
Other	3 (1.7)
**Total Income**	
10,000–24,999	4 (2.4)
25,000–34,999	11 (6.5)
35,000–49,999	11 (6.5)
50,000–74,999	22 (12.9)
>75,000	102 (60.0)
Prefer not to answer	20 (11.8)
**Physical Health Condition**	
No	65 (37.8)
Yes	107 (62.2)
**History of Anxiety**	
No	157 (90.2)
Yes	15 (8.6)
Prefer not to answer	2 (1.1)
**History of Depression**	
No	147 (84.5)
Yes	25 (13.8)
Prefer not to answer	2 (1.1)
**Diagnosis of Patient**	
Coronary Artery Disease	127 (70.2)
Arrhythmia	31 (17.1)
Congenital Heart Disease	14 (7.7)
Other	9 (5)
	**M (SD)**
**Caregiver burden (ZBI-22)**	14.32 (11.40)
**Caregiver anxiety (GAD-7)**	4.11 (4.55)
**Caregiver Depression (PHQ-9)**	4.43 (4.06)
**Anxious attachment (ECR-12)**	2.74 (1.37)
**Avoidant attachment (ECR-12)**	2.95 (1.26)

%, percentage; M, mean; n, number; SD, standard deviation.

### Mediation analyses

#### Anxiety

**Fig 2:**
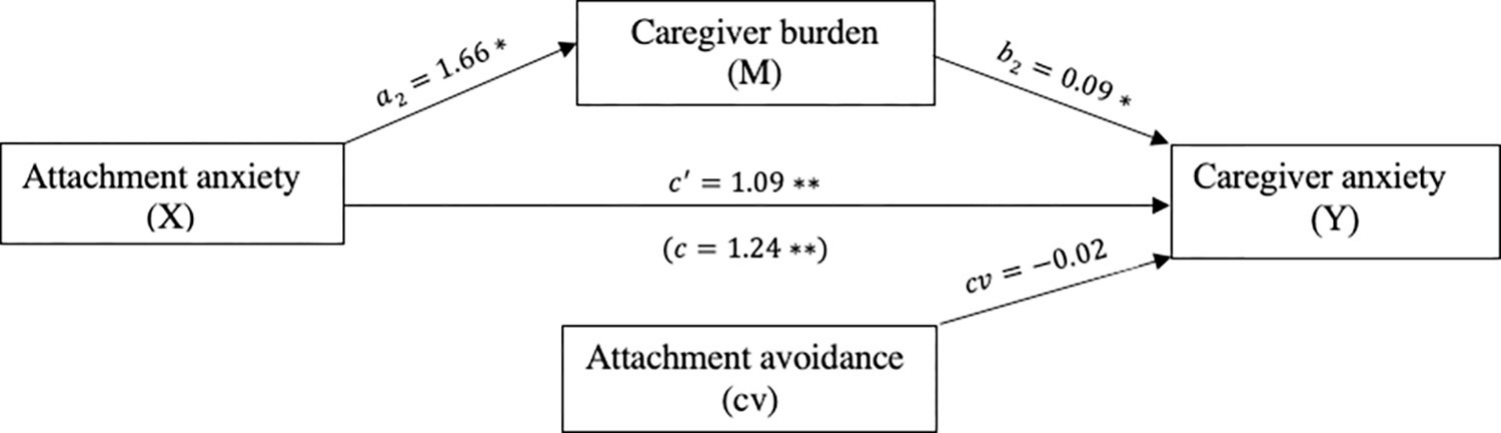
The mediating effect of caregiver burden in the relationship between attachment anxiety and caregiver anxiety. *p<0.05,**p<0.001, cv = covariate.

Attachment anxiety was significantly and positively related to caregiver anxiety (*c* = 1.24, *p*<0.001). The mediation analysis indicated that attachment anxiety was indirectly related to caregiver anxiety through its link to caregiver burden. First, as can be seen in Fig 2, caregivers with higher levels of attachment anxiety reported more caregiver burden (*a* = 1.66, *p* = 0.002), and more reported caregiver burden was subsequently related to more symptoms of anxiety (*b* = 0.09, *p* = 0.003). A 95% bias-corrected confidence interval based on 5,000 bootstrap samples indicated that the indirect effect through attachment anxiety (*ab* = 0.15) was entirely above zero (95% C.I. 0.04 to 0.29). Moreover, caregivers reported more anxiety even after taking into account the indirect effect of attachment anxiety through caregiver burden (*c*′ = 1.09, *p*<0.001). Attachment avoidance was not a significant covariate (*cv* = −0.02, *p* = 0.93).

**Fig 3:**
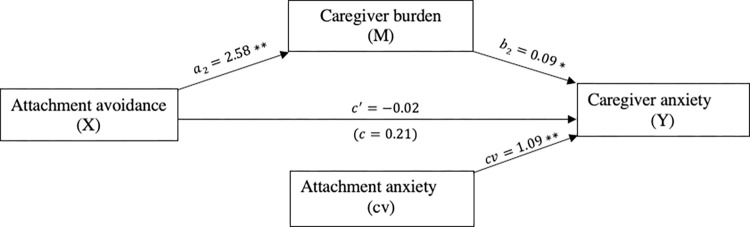
The mediating effect of caregiver burden in the relationship between attachment avoidance and caregiver anxiety. *p<0.05,**p<0.001, cv = covariate.

We did not detect a link between attachment avoidance and caregiver anxiety (*c* = 0.21, *p* = 0.37). However, the mediation analysis indicated that attachment avoidance was indirectly related to caregiver anxiety through its link to caregiver burden. First, as can be seen in Fig 3, caregivers with higher levels of attachment avoidance reported more caregiver burden (*a* = 2.58, *p*<0.001), and more reported caregiver burden was subsequently related to more symptoms of anxiety (*b* = 0.09, *p* = 0.003). A 95% bias-corrected confidence interval based on 5,000 bootstrap samples indicated that the indirect effect through attachment avoidance (*ab* = 0.23) was entirely above zero (95% C.I. 0.10 to 0.42). Caregivers with attachment avoidance did not report more anxiety when taking into account the indirect effect of attachment avoidance through caregiver burden (*c*′ = −0.02, *p* = 0.93). Of note, attachment anxiety was a significant covariate in this mediation model (*cv* = 1.09, *p*<0.001).

#### Depression

**Fig 4:**
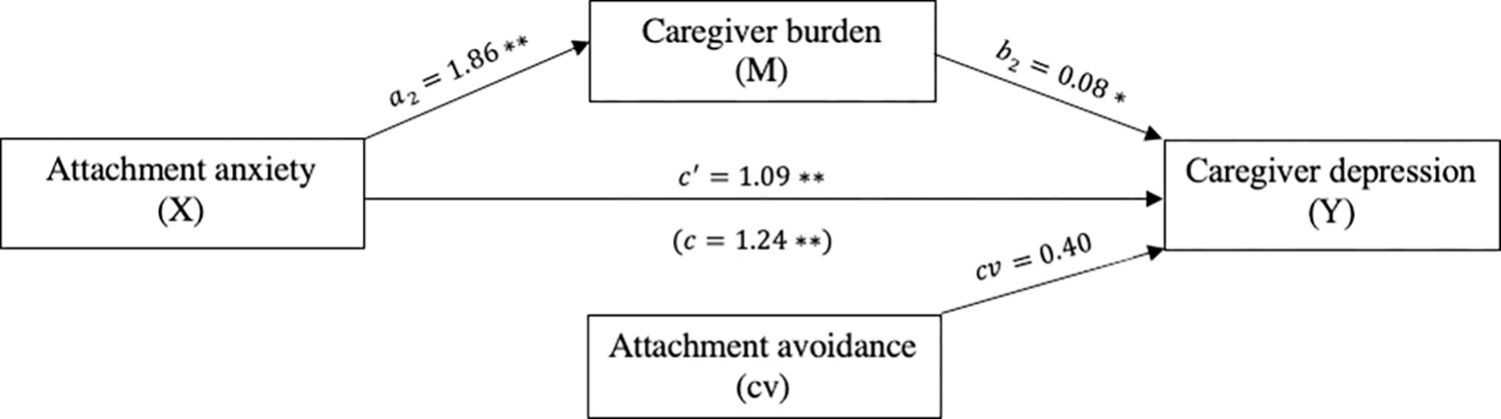
The mediating effect of caregiver burden in the relationship between attachment anxiety and caregiver depression. *p<0.05,**p<0.001, cv = covariate.

Attachment anxiety was significantly and positively related to caregiver depression (*c* = 1.24, *p*<0.001). The mediation indicated that attachment anxiety is indirectly related to depression through its relationships with caregiver burden. First, as can be seen in Fig 4, caregivers with higher levels of attachment anxiety reported more caregiver burden (*a* = 1.86, *p*<0.001), and more reported caregiver burden was subsequently related to more symptoms of depression (*b* = 0.08, *p* = 0.002). A 95% bias-corrected confidence interval based on 5,000 bootstrap samples indicated that the indirect effect through attachment anxiety (*ab* = 0.15) was entirely above zero (95% C.I. 0.05 to 0.28). Moreover, caregivers reported more depression even after taking into account the indirect effect of attachment anxiety through caregiver burden (*c*′ = 1.09, *p*<0.001). Attachment avoidance was not a significant covariate (*cv* = 0.40, *p* = 0.06).

**Fig 5:**
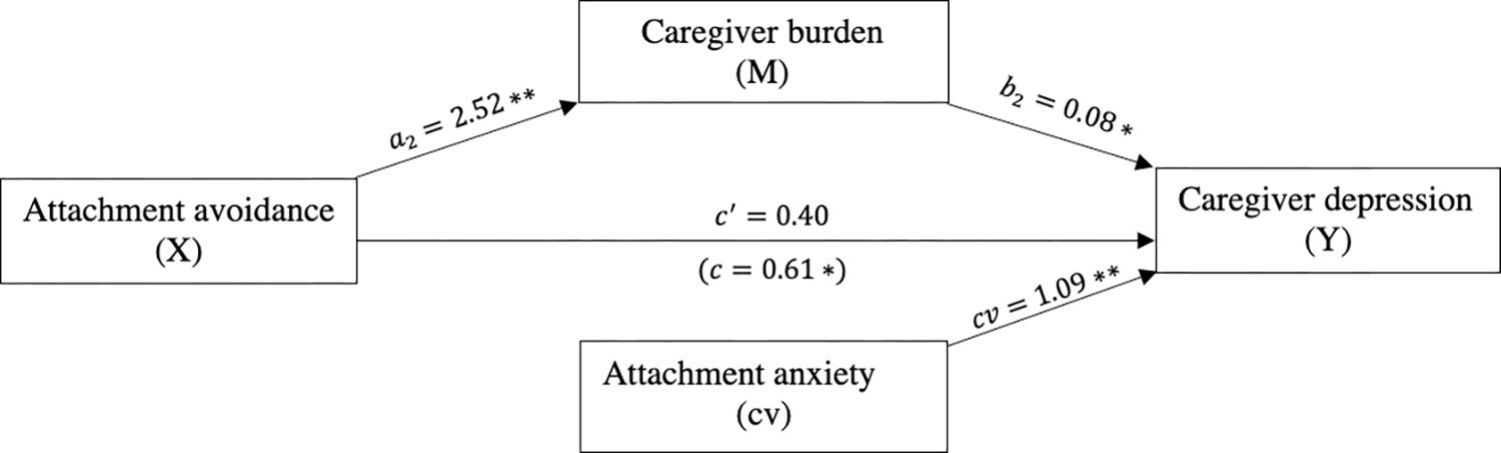
The mediating effect of caregiver burden in the relationship between attachment avoidance and caregiver depression. *p<0.05,**p<0.001, cv = covariate.

Attachment avoidance was significantly and positively related to caregiver depression (*c* = 0.61, *p* = 0.004). The mediation indicated that attachment avoidance is indirectly related to depression through its relationship with caregiver burden. First, as can be seen in Fig 5, caregivers with higher levels of attachment avoidance reported more caregiver burden (*a* = 2.52, *p*<0.001), and more reported caregiver burden was subsequently related to more symptoms of depression (*b* = 0.08, *p* = 0.002). A 95% bias-corrected confidence interval based on 5,000 bootstrap samples indicated that the indirect effect through attachment avoidance (*ab* = 0.21) was entirely above zero (95% C.I. 0.09 to 0.37). Caregivers did not report more depression after taking into account the indirect effect of attachment avoidance through caregiver burden (*c*′ = 0.40, *p* = 0.06). Of note, attachment anxiety was a significant covariate in this mediation model (*cv* = 1.09, *p*<0.001).

## Discussion

The goal of the present study was to investigate whether attachment orientation among caregivers of patients with heart disease would be indirectly related to psychological distress through its relationship with caregiver burden. To our knowledge, there is only one other study that examined the relationship among these variables. That study, however, was restricted to female caregivers and depression as the psychological outcome [48]. The present study extends our existing knowledge by exploring new possible relationships among caregiver burden, attachment, and psychological distress with a mediation analysis, a larger sample of caregivers, and the inclusion of anxiety as an outcome variable [48]. The hypothesis that attachment anxiety would increase caregivers’ experience of burden, subsequently leading to increased psychological distress was supported. Whereas, the hypothesis that attachment avoidance would limit caregivers’ experience of burden and, therefore, lower their psychological distress was not supported. Rather, attachment avoidance was related to increased caregiver burden and a subsequent increase in psychological distress. In order to understand these findings, it is important to consider the emotional experiences of people with attachment insecurities, as this may affect their perception of their caregiving role and, thereby, contribute to their psychological distress [25,26,37,38,45–47].

In regard to caregivers with higher levels of attachment anxiety, they are likely to engage in “distress-exacerbating mental rumination–moody pondering, or thinking anxiously or gloomily about a threatening event and paying more attention to distress-eliciting stimuli” [38,59–62]. This may cause them to perceive their caregiving role as more burdensome than someone with lower levels of attachment anxiety [37,45–47]. Thus, attachment anxiety would add to the psychological toll of caregiver burden which, even in the absence of attachment anxiety, is related to an increase in psychological distress [25,48].

In regard to attachment avoidance, it was hypothesized that caregivers with this attachment orientation would suppress negative affect related to caregiving, thereby allowing them to engage in this role while reporting less negative affect than individuals with higher levels of attachment anxiety or attachment security. However, this hypothesis was not supported, and the current data showed that caregivers with attachment-related avoidance reported increased levels of caregiver burden which was, in turn, related to increased levels of psychological distress. It has been shown in the literature that deactivating strategies break down when individuals with attachment-related avoidance endure too much distress, meaning that they have difficulty suppressing the negative affect they have been experiencing internally, such that it becomes more apparent [63–65]. Indeed, this fragility contributes to the reason that deactivating strategies are considered secondary attachment strategies and less effective methods of emotion regulation than the primary attachment strategies used by those with a secure sense of attachment [66].

In the present study, it is possible that caregivers were not only threatened by the loss of their primary attachment figure as a result of the heart disease, but they also faced myriad of practical challenges related to their caregiving role [5,11,13,15]. The data showed that caregivers with attachment-related avoidance did not report more psychological distress without consideration of their caregiving burden but they did report more psychological distress when caregiver burden is taken into account. These relationships demonstrate that the additional burden of the caregiving role may render the deactivating strategies ineffective; they simply cannot escape from the duties and, therefore, distress.

Vilchinsky et al. [48] hypothesized, using an all-female caregiver sample and moderation analysis, that the relationship between caregiver burden and depression would be weakened by higher levels of attachment-related avoidance [48].This hypothesis assumed that caregivers with higher levels of attachment avoidance may view caregiving as a threat to maintaining emotional distance from their attachment figure, causing them to supress negative affect and, thereby weakening the relationship between caregiver burden and symptoms of depression. However, their findings did not support this hypothesis. The authors speculated that “experiencing a partner’s life-threatening illness—and being placed in the role of caretaker—may make it more difficult for caregivers who are high on avoidant attachment to suppress their distress and stay emotionally detached [which] is congruent with previous findings showing that under intense stress, deactivating strategies [become] less effective. . .” [48]. The findings in the current study provide some statistical evidence to corroborate Vilchinsky et al.’s [48] explanation as to why attachment-related avoidance was an insignificant moderator. Nonetheless, further research that addresses the limitations of the current study are needed to provide more evidence that caregiver burden renders deactivating strategies ineffective.

Limitations of this study should be considered when interpreting the results. Mediation analyses are multiple regression analyses where the dependent and independent variables are chosen based on theory; as such, assumptions of causality cannot be made [57]. Despite cross-sectional data being used in past literature to investigate mediational relationships in a health context [67,68], this type of data inhibits causal inferences and longitudinal data would enhance future research [69,70]. Further, Kane and Ashbaugh [57] suggest that an experimental design that manipulates variables is needed in order to properly determine causality.

There are also restrictions on the generalizability of the findings due to the homogeneity of sample characteristics (i.e., caregivers were mostly white females with an annual household income of more than $75,000). Similarly, 70% of the patients to whom the caregivers were providing care had coronary artery disease. Although the sample was more diverse than the previous study [48] investigations of caregivers responding to the needs of patients with various cardiac diagnoses would be a welcome addition to the literature. Our sample also reported relatively secure attachment. Individuals with a more stable sense of attachment have more effective affect-regulation strategies, which would explain the low mean scores on caregiver burden and distress [37,38]. Future studies with caregivers with higher attachment insecurity and psychological distress are warranted. The lower scores on caregiver burden and distress may also be explained by the severity of and length of time since the cardiac event. The necessary data to analyze these relationships was not collected as a part of this study but should be considered in future studies. Finally, our participants were partners of patients enrolled in cardiac rehabilitation. Patients who attend cardiac rehabilitation are known to be more affluent and a part of well-adjusted relationships (and possibly, then their partners experience lower burden or distress) as compared to those who do not engage in these programs [71,72]. Despite these limitations, the large sample size and novelty of the study (i.e., only one previous study focused on these variables in the relevant sample) are notable strengths.

### Clinical implications

The findings of the present study have important clinical implications. Current practice predominantly excludes the caregiver from formal programs, despite increasing attention to the health and wellbeing of cardiac caregivers alongside the patient in rehabilitation [73,74]. Interventions should target caregivers reporting burden and attachment insecurity to potentially lessen caregiver distress as they support their partners with heart disease. The findings in this study suggest that couple-based intervention programs that use an attachment lens might benefit the health and well-being of both patient and partner. In fact, burgeoning research supports the need and effectiveness of this form of intervention in a cardiac population [73–75]. In fact, a qualitative study found that couples desired interventions for relationship enhancement and the opportunity to share their experience of cardiac-related events with peers [75]. These areas were included in an attachment-based intervention that demonstrated positive clinical and statistical mental health and quality of life changes in patients with heart disease and their partners [73]. In summary, the findings reported herein have important theoretical and clinical implications. As noted, caregiver burden mediated both the relationships between attachment anxiety and psychological outcomes and attachment avoidance and psychological outcomes. Future research exploring interventions targeting attachment orientation is warranted.
